# Association between Fish Consumption during Pregnancy and Maternal and Neonatal Outcomes: A Statistical Study in Southern Italy

**DOI:** 10.3390/jcm13072131

**Published:** 2024-04-07

**Authors:** Angela Alibrandi, Agata Zirilli, Maria Le Donne, Carlo Giannetto, Maurizio Lanfranchi, Angelina De Pascale, Chiara Politi, Giosuè Giordano Incognito, Alfredo Ercoli, Roberta Granese

**Affiliations:** 1 Department of Economics, University of Messina, 98100 Messina, Italy; aalibrandi@unime.it (A.A.); azirilli@unime.it (A.Z.); carlo.giannetto@unime.it (C.G.); maurizio.lanfranchi@unime.it (M.L.); angela.depascale@unime.it (A.D.P.); 2 Unit of Gynecology and Obstetrics, Department of Human Pathology of Adults and Developmental Age, “G. Martino” University Hospital, 98100 Messina, Italy; mledonne@unime.it (M.L.D.); pltchr89h57i754o@studenti.unime.it (C.P.); aercoli@unime.it (A.E.); 3 Department of General Surgery and Medical Surgical Specialties, University of Catania, 95123 Catania, Italy; giosuegiordano.incognito@studium.unict.it; 4 Department of Biomedical and Dental Sciences and Morphofunctional Imaging, “G. Martino” University Hospital, 98100 Messina, Italy

**Keywords:** fish consumption, pregnancy outcome, maternal complications, neonatal weight, neonatal head circumference

## Abstract

**Background:** This research aimed to evaluate the association between the monthly consumption of fish (differentiated by type) and both gestational and neonatal outcomes. **Methods:** Women who were admitted for delivery in the last 6 months of 2023 were prospectively included and divided according to type of fish consumed (based on DHA and mercury content) and frequency of consumption. Neonatal outcomes included weight, length, head circumference, and 1st and 5th minute Apgar scores. Maternal outcomes were threats of abortion, preterm birth, gestational diabetes and hypertension, cesarean section, and differential body mass index (BMI). **Results:** Small-size oily fish with high DHA and low mercury content (type B fish) consumption was positively associated with neonatal weight and head circumference, and less weight gain in pregnancy. It was also significantly associated with lower incidences of gestational diabetes and hypertension, and cesarean section. Correlation between differential BMI and monthly consumption of fish resulted in statistical significance, especially in type B fish consumers. **Conclusions:** The consumption of type B fish was significantly associated with increased neonatal weight and head circumference and better maternal outcomes.

## 1. Introduction

Today’s consumers are increasingly interested in the relationship between food and health, as food serves not only as a means to meet the body’s nutritional needs but also as a tool for preventing diseases [1]. “Functional foods” are considered to be those food products that provide essential nutrients and potentially have a positive impact on human health [2]. For example, the consumption of fish is a significant benefit in terms of preventing overweight and obesity and may have a protective effect against certain types of cancer and cardiovascular, autoimmune, neurodegenerative, and ocular diseases [3,4,5,6,7,8,9,10,11,12,13,14,15,16,17]. Fish are rich in nutrients that are beneficial for pregnancy progression, as well as for fetal growth and development. These include protein, long-chain omega-3 polyunsaturated fatty acids (LCPUFAs), such as docosahexaenoic acid (DHA), selenium, iodine, and vitamin D [18,19,20,21,22]. However, studies on the associations between maternal fish consumption and both gestational and neonatal outcomes are contrasting [23,24,25,26,27]. This discrepancy has been partly attributed to the different content in certain substances among different types of fish. For instance, fatty fish contains larger amounts of beneficial LCPUFA and, similar to shellfish, larger amounts of pollutants, such as mercury and other heavy metals [28,29,30]. Thus, this study aims to evaluate the association between the usual monthly consumption of fish (differentiated by type) and both neonatal and maternal outcomes. In a research study conducted in Italy by Le Donne et al. in 2016 [31] on a sample of women giving birth, it was found that small oily fish consumption should be favored over other types of fish.

The added value of this research, in the context of the existing literature, is to connect aspects linked to habitual fish consumption (based on frequency and type) to the health of pregnant mothers and unborn children.

## 2. Materials and Methods

This single-center prospective study was carried out by administering a questionnaire about fish consumption and collecting data from the medical records of the pregnant women who were admitted to the Unit of Obstetrics and Gynecology of “G. Martino” University Hospital, Messina, Italy for delivery from July 2023 to December 2023. Data retrieved included family history, personal and midwife history, lifestyle, monthly fish habitual consumption, and type of fish consumption. The following requirements had to be met in order to be eligible: to be a native-born, non-immigrant Caucasian woman living in the province; to have a normal weight and stable Mediterranean dietary habits; if they ate fish, to eat it unfried at home; to have a physiological pregnancy; to consume DHA daily in a dose of 200 mg; to give birth to a singleton live baby. A history of drug or alcohol abuse, smoking, a personal or family history of diabetes, a personal history of recurrent miscarriages, diabetes or preeclampsia in previous pregnancies, thrombophilia, anemia, chronic hypertension, lower urinary tract infections, any other previous or current non-obstetric or obstetric pathology that could have altered the significance of the results, any indication for an elective cesarean section, undergoing treatment with any medications, and not consuming any DHA supplements were the exclusion criteria.

Dietary assessment was carried out at the first visit through a face-to-face interview using an Italian food frequency questionnaire [32] with the assistance of color photographs to show portion size [33].

Adherence to the Mediterranean diet [34] was also assessed.

The monthly frequency of maternal fish intake was categorized as follows: 0 times (never), 1–4 times (occasionally), 5–8 times (regularly), and more than 8 times (frequently).

Four types of fish were considered: (A) large-size oily fish with both high DHA and mercury content (tuna, swordfish), (B) small-size oily fish with high DHA and low mercury content (mackerel, salmon, anchovy, garfish, spatula, sardine), (C) lean fish with low DHA and medium mercury content (sea gilt-head bream, sea bass, cod, sea bream, perch), and (D) shellfish with low DHA and low mercury content [35,36,37].

Neonatal and gestational outcomes were investigated. Gestational outcomes included length of gestation, pregnancy complications: preterm birth, cesarean section, gestational hypertension (PIH), gestational diabetes mellitus (GDM), and threat of abortion. Neonatal outcomes were height (NH), weight (NW), head circumference (NHC), and 1st minute and 5th minute Apgar scores.

All the designs, analysis, interpretation of data, drafting, and revisions comply with the Reporting of Observational Studies in Epidemiology (STROBE) [38], drawn up by the Enhancing the QUAlity and Transparency Of health Research (EQUATOR) network [39] and we approved by the Institutional Review Board (IRB) of the University Hospital in which it was performed.

All volunteers signed a consent form to declare their voluntary agreement with all procedures implicit in their involvement. Participants were informed that their participation could be voluntarily interrupted at any time without any consequence to the woman or the quality of her health care. All information obtained from volunteers was treated as confidential and anonymous, respecting privacy.

Categorical variables were expressed as absolute frequencies and percentages, and numerical variables as mean and standard deviation. The parametric approach [40] was applied in the data analyses, as the Kolmogorov–Smirnov test provided evidence of normal distribution for most of the analyzed variables.

In order to identify the existence of possible predictors of neonatal outcomes (weight, length, head circumference, and 1st minute and 5th minute Apgar scores), some multivariable linear regression models were estimated [41,42]. The analyzed covariates were age, body mass index (BMI) (at the end of pregnancy), duration of pregnancy, preterm birth, gestational diabetes, gestational hypertension, threats of abortion, monthly fish habitual consumption, and type A, B, C, and D fish consumption. The results were expressed as B regression coefficient, 95% confidence interval (C.I.), and *p*-value.

In addition, multivariable binary logistic regression models [43] were estimated to identify significant predictors of maternal gestational complications (preterm birth, gestational diabetes, gestational hypertension, cesarean section, and threats of abortion). In particular, the relationship of the following covariates was tested: age, BMI at baseline, type A, B, C, and D fish consumption. The results were expressed as Odds Ratio (OR), 95% C.I., and *p*-value.

Finally, after checking the Gaussianity of the differential BMI distribution (considered as the difference between the BMI at the end of pregnancy and the pre-pregnancy BMI), another linear regression model was estimated to identify predictive factors of this maternal outcome. The Pearson correlation test was applied to assess the possible interdependent relationship between the differential BMI of women at the end of pregnancy and the monthly frequency of fish consumption, both in the sample of fish consumers and, specifically, according to the type of fish.

A *p*-value < 0.05 was considered statistically significant and is reported in bold in all tables.

All statistical analyses were performed by means of the SPSS for Windows package, version 22.

## 3. Results

### 3.1. Clinical Variables According to the Fish Type Consumption

**Figure 1:**
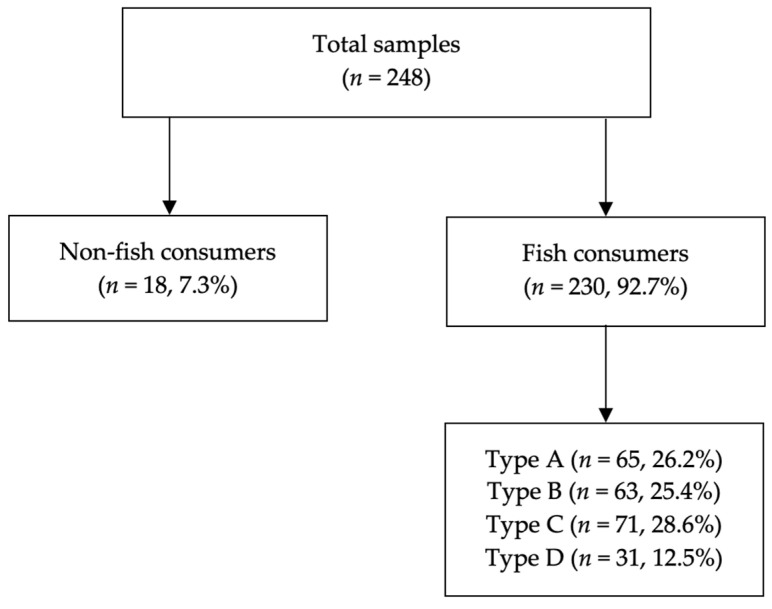
Women included according to fish type consumption.

The descriptive statistics of the clinical variables are presented in Table 1. The description was carried out for all respondents in the subpopulation of fish consumers and non-fish consumers, and, also, based on the specific type of fish consumed (type A, B, C, and D). A total of 248 women were included in the study, with 18 women (7.3%) non-fish consumers, 40 women (16.1%) consuming fresh fish 1–4 times per month (occasionally), 144 women (58.1%) consuming 5–8 times per month (regularly), and 46 women (18.5%) more than 8 times per month (frequently). Of these, 230 (92.7%) fish consumers, the number of consumers of type A and B fish was almost coincidental (65 and 63 women, respectively), while 71 women and 31 women were included in groups C and D, respectively (Figure 1). The average age of all women at enrolment was 31.7 ± 5.5 years. Regarding obstetric history, 74 women were nulliparous and 174 were multiparous; 44 women had had at least one abortion.

### 3.2. Neonatal Outcomes

**Figure 2:**
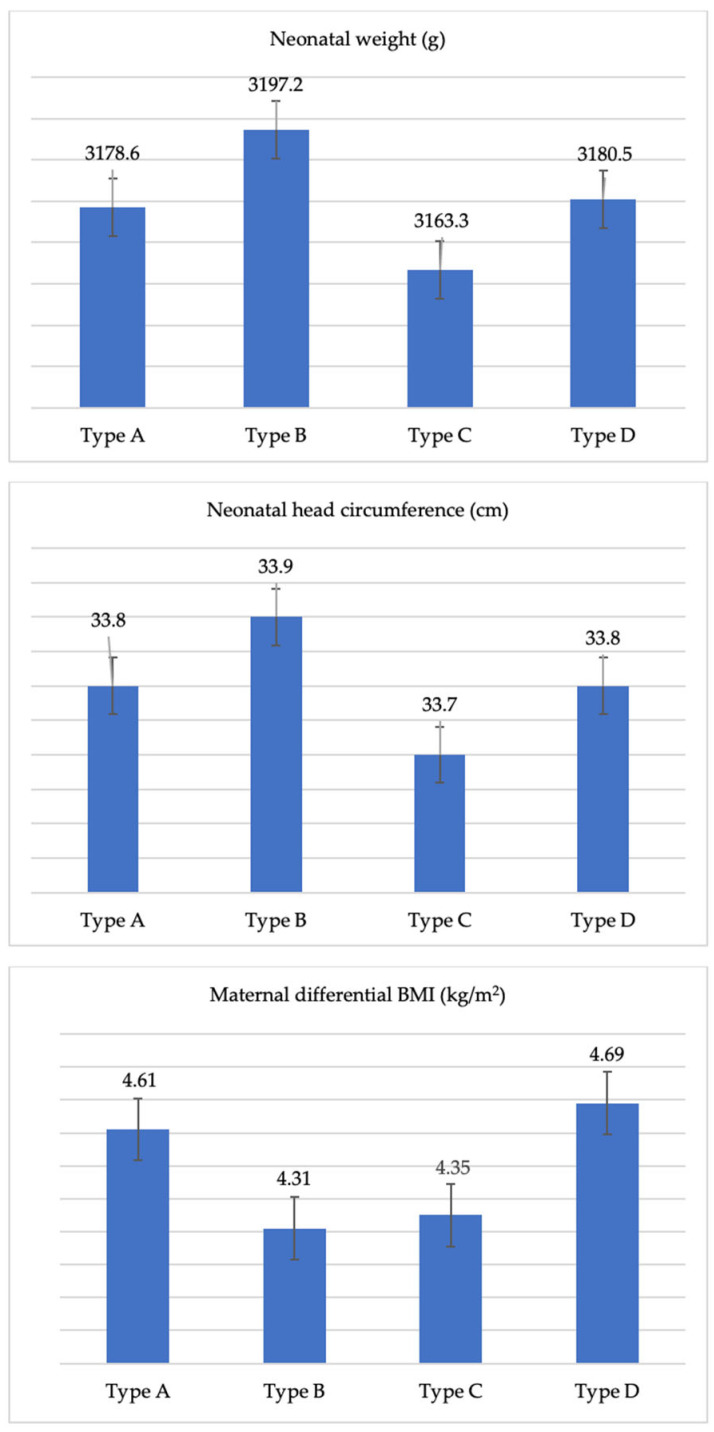
Neonatal weight, neonatal head circumference and maternal differential body mass index according to maternal consumption of fish by type. Values are expressed as mean ± standard deviation.

The multivariable linear regression model highlighted that significant predictors of NW were the duration of the pregnancy (b = 1.69, *p* = 0.035); consequently, preterm birth was associated with a lower weight of the newborn (b = −0.15, *p* = 0.015) (Table 2). Of interest, the consumption of type B fish was significantly associated with a greater weight of the unborn child (b = 210.63; *p* = 0.031). Furthermore, consuming type B fish was also positively associated with another neonatal outcome, the NHC (b = 0.70, *p* = 0.013). The positive sign of the coefficient showed that the consumption of type B fish was associated with a higher NHC and, therefore, with greater neonatal development (Table 2; Figure 2). The same model, estimated for NH and 1st minute and 5th minute Apgar scores, did not provide any statistical significance.

### 3.3. Maternal Outcomes

Focusing on gestational complications, estimation of the binary logistic regression model showed that the consumption of type B fish was significantly associated with lower occurrences of GDM (OR = 0.19, *p* = 0.021), PIH (OR = 0.01, *p* = 0.010), and cesarean section (OR = 0.54, *p* = 0.029), while its relationship with preterm birth and threats of abortion was not significant (*p* = 0.0174 and *p* = 0.078, respectively) (Table 3).

#### Maternal Outcomes: Differential Body Mass Index

Focusing further attention on the physical health of mothers, the estimate of the linear regression model for the “differential BMI” outcome (obtained as the difference between the BMI at the end of pregnancy and the BMI at the beginning of pregnancy) showed that the consumption of type B fish was significantly associated with a lower weight gain during pregnancy (b = −0.61, *p* = 0.029) (Table 4; Figure 2). Even GDM significantly reduces the differential BMI (b = −1.06; *p* = 0.038). This aspect justifies the negative sign of the regression coefficient (Table 4).

Finally, the interdependence relationship between the differential BMI and the monthly consumption of fish was analyzed, both in the entire sample and in the subgroup of fish consumers, and, more specifically, in the subgroups of pregnant women according to the specific type of fish consumed (Table 5). The results obtained showed that the frequency of fish consumption generally reduced the weight gain of pregnant women and, specifically, the consumption of type B fish determined a significant reduction in terms of differential BMI.

## 4. Discussion

This study aims to evaluate the correlation between the usual monthly consumption of fish (differentiated by type) and both neonatal and maternal outcomes.

The results of the study confirm that this association is linked to the type of fish consumed, which in turn relates to the levels of subnutrients, such as LCPUFAs, and pollutants present. It is known that large and small oily fish are rich in DHA, whereas lean fish and shellfish have lower levels of this nutrient. Conversely, large oily fish tend to have higher concentrations of contaminants such as mercury, with lean fish having lesser amounts when compared to small oily fish and shellfish [36,37]. Additionally, mussels are known to gather significant quantities of metals and persistent pollutants [44]. The result of interest for the purposes of this research was the significance of the consumption of type B fish, as it was significantly associated with greater NW and NHC, important indicators of infant health. Another significant predictor of NW was the duration of the pregnancy because longer durations lead, as is to be expected, to a higher weight of the unborn child and, consequently, the condition of full-term birth. These data are in agreement with a previous study by Le Donne et al. [31], in which it was found that the prevalent consumption of small blue fish (less exposed to the risk of accumulating contaminants and richer in DHA) was positively associated with NW and NHC. In contrast, the prevalent consumption of tuna and swordfish (also rich in DHA but high in pollutants) or lean fish and/or shellfish (low in DHA) was negatively associated with the considered parameters of the unborn child. These results also align with those of Ramon et al. [45], who suggested that differences in neonatal outcomes between female consumers of small and large fish high in fatty acids were due to the balance between beneficial nutrients, mainly DHA, and toxic substances present in each species. Surprisingly, the data of the present study did not show any statistical significance between monthly fish consumption and neonatal outcomes. This would reinforce the hypothesis that the type of fish consumed plays a more significant role in these outcomes than the consumption of fish alone. However, Heppe et al. [46] found no consistent associations of total-fish, lean-fish, or fatty-fish consumption with fetal growth characteristics at birth in a cohort of pregnant women in The Netherlands.

Regarding gestational outcomes, the consumption of small-size oily fish was associated with a reduced occurrence of GDM and PIH, as well as with fewer cesarean sections and less weight gain. Even GDM, in truth, significantly reduced the differential BMI; in fact, as is to be expected, those who had this pregnancy pathology followed a stricter and more rigorous diet, so they gained fewer kilos at the end of the pregnancy. The findings on the relationship between differential BMI and monthly fish consumption have reinforced this hypothesis, showing that the frequency of fish consumption generally reduced weight gain. The positive correlation of type B fish with PIH is in agreement with the data found by Oken et al. [47], who reported a lower risk in women with a higher intake of LCPUFA. Furthermore, women who consumed type B fish and were giving birth also had significantly reduced rates of cesarean sections, with which some gestational complications such as PIH and GDM are positively correlated.

Few other studies have examined the relationship between fish consumption and gestational and neonatal outcomes in terms of both frequency of fish intake and/or type of fish consumed. This study was limited to evaluating such findings on the newborn. However, a study conducted in Denmark by a group of Danish researchers analyzed the correlation between fish consumption during pregnancy and lactation duration in relation to the achievement of specific postnatal neurodevelopmental milestones. Pregnant women who consume one or more servings of fish per week have superior offspring neurodevelopmental outcomes, according to an extensive review of observational cohort studies [18,48,49]. Other findings have highlighted that higher fish consumption by pregnant women and a longer duration of breastfeeding are independently correlated with better results in cognitive tests conducted on the child [48]. It was also reported that in pregnant women stable consumption of oily fish (which is enriched in LCPUFA) protects from postpartum thyroiditis (PPT) and postpartum depression, while stable consumption of swordfish (which is enriched in pollutants) favors PPT [17,21,50]. The serum concentration of antithyroid antibodies was lower compared with pregnant women consuming predominantly swordfish, resulting in a reduced risk of developing PPT and postpartum depression.

Consumption of fish has an undoubted protective effect on various diseases [3,4,5,6,7,8,9,10,11,12,13,14,15,16,17]. For this reason, a paper by the American Heart Association (AHA) [51] and a joint paper published in 2009 by the Food and Agriculture Organization of the United Nations (FAO) and the World Health Organization (WHO) [52] recommend consuming two meals based on seafood each week. The European Food Safety Agency (EFSA), based on advice from leading international scientific societies, suggests an intake of LCPUFA, which some fish are rich in, at a dose of 250 mg per day [53]. Moreover, in addition to eating seafood twice a week, one of those meals should include fatty fish, according to the Sixth Joint Task Force of the European Society of Cardiology and Other Societies on Cardiovascular Disease Prevention in Clinical Practice [4]. The beneficial effect represented by fish is, in fact, related to its omega-3 LCPUFA content. Furthermore, the usefulness of recommending high intakes of *n*-3 fatty acid-rich fish (as opposed to total seafood) remains questionable, especially in light of contradictory observational study findings and risk assessments regarding potential contaminants present in large fatty fish [54,55]. According to specific meta-analytic results, eating fatty fish is inversely related to the incidence of coronary heart disease and attributable mortality, but these relationships did not hold true when lean fish consumption was taken into account [56].

During pregnancy, the Food and Drug Administration (FDA) advised women to eat 8–12 ounces (227–340 g), namely 2–3 servings of a variety of fish each week, avoiding four types of fish with the highest content of mercury (tilefish from the Gulf of Mexico, shark, swordfish, and king mackerel), and preferring those types of fish with the lowest content (salmon, shrimp, canned fish, catfish, and cod) [36]. Moreover, the amounts of EPA and DHA should be increased to at least 300 mg daily, including at least 200 mg of DHA [57]. In fact, DHA, which the human body cannot synthesize, is necessary for the development of the brain and retina of the unborn child [18]. Additionally, some papers reported that it reduces the risk of gestational hypertension, premature birth, intrauterine growth retardation, and postpartum depression in the mother [18,19,20,21,22]. However, only 46 out of 248 (18.5%) pregnant women in the present study reported “frequent” fish consumption (up to 8 times monthly), following the recommended doses by the FDA.

A strength of this research is that detailed information on the consumption of different fish types was prospectively collected, enabling separation of the analyses. Also, from the questionnaires, information on many potential confounding variables was extensively collected. For example, only women who took the same DHA supplementation (200 mg daily) were included to ensure that differing supplementation not derived from fish did not act as a confounding factor in the results.

Limitations include the single-center observational study design, which may impact the generalizability of its conclusions and prevent drawing causal inferences. Additionally, the relatively small sample size and reliance on self-reported dietary information could introduce bias, potentially affecting the accuracy and applicability of the results. These factors, combined with the study’s regional focus, suggest that, while the findings are significant, they should be interpreted with caution when considering their relevance to diverse populations. However, despite the collection of many potential confounding variables, there could be additional confounders and covariates that might account for the observed associations. Finally, the study’s brief follow-up period restricts the ability to assess the long-term outcomes on both mothers and, above all, children associated with fish consumption.

## 5. Conclusions

The consumption of type B fish (mackerel, salmon, anchovies, etc.) significantly increased the NW and NHC and was significantly associated with the BMI of the mother (i.e., those who consumed type B fish gained fewer kilos at the end of pregnancy). Complications (GDM, PIH, and cesarean section) in pregnancy were also significantly reduced in those who consumed type B fish. Conversely, the monthly fish consumption seemed unrelated to neonatal outcomes. Further prospective, multicentric studies with more extended follow-up periods are necessary to confirm these findings and analyze the long-term outcomes on infants.

## Figures and Tables

**Table 1:** Descriptive statistics of the clinical variables according to fish type consumption.

	All	Fish Consumers	Non-Fish Consumers	Type A	Type B	Type C	Type D
	(*n* = 248)	(*n* = 230)	(*n* = 18)	(*n* = 65)	(*n* = 63)	(*n* = 71)	(*n* = 31)
Age (years)	31.7 ± 5.5	31.8 ± 5.3	31.9 ± 6.1	31.0 ± 5.8	32.2 ± 5.6	32.1 ± 5.4	31.4 ± 5.9
BMI at the beginning of pregnancy (kg/m^2^)	24.1 ± 4.8	23.9 ± 4.8	24.9 ± 5.1	24.3 ± 5.1	24.4 ± 5.0	23.9 ± 4.9	24.3 ± 5.5
BMI at the end of pregnancy (kg/m^2^)	28.7 ± 4.6	28.6 ± 4.5	29.9 ± 5.3	29.1 ± 4.6	28.8 ± 4.5	28.3 ± 4.6	29.1 ± 5.0
Duration of pregnancy (days)	272.2 ± 19.2	272.1 ± 19.9	273.4 ± 11.9	272.8 ± 11.8	271.9 ± 23.7	271.4 ± 22.8	271.3 ± 28.2
1st minute Apgar score	8.8 ± 1.0	8.9 ± 1.0	8.7 ± 1.2	8.8 ± 1.0	8.9 ± 0.9	8.8 ± 0.9	8.8 ± 1.1
5th minute Apgar score	9.7 ± 0.5	9.7 ± 0.5	9.5 ± 0.7	9.7 ± 0.6	9.8 ± 0.5	9.7 ± 0.6	9.8 ± 0.5
NW (mm)	3174.2 ± 472.6	3179.1 ± 459.2	3131.2 ± 588.6	3178.6 ± 448.8	3197.2 ± 464.9	3163.3 ± 461.3	3180.5 ± 399.5
NL (mm)	48.4 ± 2.7	48.4 ± 2.7	49.1 ± 2.8	48.4 ± 2.6	48.5 ± 3.1	48.4 ± 2.6	48.3 ± 1.9
NHC (mm)	33.9 ± 2.0	33.9 ± 2.0	34.3 ± 1.7	33.8 ± 1.3	33.9 ± 2.3	33.7 ± 2.3	33.8 ± 1.4
Preterm birth	22 (8.9%)	20 (8.7%)	2 (11.1%)	5 (7.7%)	5 (7.9%)	7 (9.8%)	3 (9.7%)
Gestational diabetes	13 (5.2%)	11 (4.8%)	2 (11.1%)	4 (6.2%)	2 (3.2%)	4 (5.26)	1 (3.2%)
Gestational hypertion	10 (4%)	6 (2.6%)	4 (22.2%)	2 (3.1%)	1 (1.6%)	2 (2.8%)	1 (3.2%)
Cesarean section	11 (44.8%)	101 (43.9%)	10 (55.5%)	30 (46.2%)	27 (42.8%)	31 (43.7%)	13 (41.9%)
Threats of abortion	36 (14.5%)	33 (14.3%)	3 (16.7%)	8 (12.3%)	11 (17.6%)	8 (11.3%)	6 (19.4%)
Monthly fish consumption:							
Never	18 (7.3%)	0 (0%)	18 (100%)	0 (0%)	0 (0%)	0 (0%)	0 (0%)
Occasionally (1–4 times a month)	40 (16.1%)	40 (17.4%)	0 (0%)	13 (15.6%)	7 (11.1%)	14 (19.7%)	6 (19.4%)
Regularly (5–8 times a month)	144 (58.1%)	144 (62.6%)	0 (0%)	37 (57%)	45 (71.4%)	41 (57.7%)	21 (67.7%)
Frequently (>8 times a month)	46 (18.5%)	46 (20%)	0 (0%)	15 (27.4%)	11 (17.5%)	16 (22.5%)	4 (12.9%)

Abbreviations: BMI, body mass index; *n*, number; NHC, neonatal head circumference; NL, neonatal length; NW, neonatal weight. For the numerical variables, the mean ± standard deviation (SD) was reported, and for the qualitative variables the absolute and percentage frequencies.

**Table 2:** Multivariate linear regression model for neonatal weight and head circumference.

Independent Variables	NW				NHC			
	B	95% C.I. for B		*p*-Value	B	95% C.I. for B		*p*-Value
Age (years)	7.47	1.76	14.70	0.392	0.04	0.01	0.09	0.083
BMI at the end of pregnancy (kg/m^2^)	12.41	4.77	29.58	0.155	0.05	0.00	0.10	0.055
Duration of pregnancy (days)	1.69	1.30	5.30	0.035	1.02	0.94	1.27	0.057
Preterm birth	−0.15	−0.60	−0.09	0.015	−0.39	−1.40	0.61	0.437
Gestational diabetes	158.72	108.64	202.08	0.361	0.32	0.07	1.31	0.520
Gestational hypertension	14.06	3.78	58.89	0.535	0.39	0.05	1.83	0.592
Threats of abortion	9.00	1.77	23.96	0.939	−0.02	−0.68	−0.06	0.953
Monthly fish consumption	21.15	5.42	18.12	0.155	0.04	0.01	0.15	0.406
Type A fish consumption	105.16	69.95	280.26	0.236	0.14	0.16	0.36	0.584
Type B fish consumption	210.63	119.92	301.35	0.031	0.70	0.15	1.25	0.013
Type C fish consumption	76.20	41.04	153.44	0.396	0.47	0.20	0.94	0.072
Type D fish consumption	57.70	33.28	120.89	0.523	0.48	0.20	0.74	0.070

Abbreviations: BMI, body mass index; C.I., confidence interval; NHC, neonatal head circumference; NW, neonatal weight.

**Table 3:** Multivariate logistic regression model for gestational complication.

	Gestational Diabetes Mellitus			
Variables	OR	95% C.I. for OR		*p*-Value
Age (years)	1.02	0.91	1.14	0.750
BMI at baseline (kg/m^2^)	1.04	0.92	1.16	0.545
Type A fish consumption	1.11	0.33	3.76	0.871
Type B fish consumption	0.19	0.05	0.78	0.021
Type C fish consumption	2.30	0.66	7.98	0.190
Type D fish consumption	0.64	0.16	2.54	0.529
	**Gestational hypertension**			
Age (years)	1.03	0.87	1.22	0.717
BMI at baseline (kg/m^2^)	1.46	1.22	1.74	0.013
Type A fish consumption	0.84	0.14	5.00	0.850
Type B fish consumption	0.01	0.00	0.32	0.010
Type C fish consumption	1.03	0.18	5.75	0.974
Type D fish consumption	0.97	0.14	6.70	0.979
	**Pre-term birth**			
Age (years)	0.90	0.77	1.05	0.188
BMI at baseline (kg/m^2^)	1.12	0.95	1.32	0.184
Type A fish consumption	0.29	0.05	1.82	0.188
Type B fish consumption	0.22	0.02	1.96	0.174
Type C fish consumption	1.91	0.38	9.54	0.429
Type D fish consumption	0.80	0.13	4.69	0.800
	**Cesarean section**			
Age (years)	1.04	0.99	1.09	0.132
BMI at baseline (kg/m^2^)	1.07	1.01	1.13	0.023
Type A fish consumption	1.14	0.66	1.99	0.641
Type B fish consumption	0.54	0.31	0.94	0.029
Type C fish consumption	1.38	0.81	2.37	0.241
Type D fish consumption	1.09	0.62	1.89	0.772
	**Threats of abortion**			
Age (years)	0.92	0.83	1.02	0.144
BMI at baseline (kg/m^2^)	0.81	0.69	0.96	0.015
Type A fish consumption	0.84	0.29	2.41	0.749
Type B fish consumption	0.79	0.26	2.09	0.078
Type C fish consumption	0.52	0.18	1.51	0.232
Type D fish consumption	1.14	0.40	3.28	0.797

Abbreviations: BMI, body mass index; C.I., confidence interval, OR, odds ratio.

**Table 4:** Multivariate linear regression model for differential body mass index.

Variables	B	95% C.I. for B		*p*-Value
Age (years)	−0.01	−0.05	0.04	0.833
Duration of pregnancy (days)	0.01	0.00	0.03	0.112
Preterm birth	−0.36	−1.32	0.60	0.461
Gestational diabetes	−1.06	−2.06	−0.06	0.038
Gestational hypertension	0.48	−1.28	2.23	0.592
Threats of abortion	0.63	−0.10	1.37	0.092
Monthly fish consumption	−0.01	−0.12	0.10	0.883
Type A fish consumption	0.20	−0.34	0.74	0.464
Type B fish consumption	−0.61	−1.15	−0.06	0.029
Type C fish consumption	−0.33	−0.86	0.19	0.213
Type D fish consumption	0.22	−0.32	0.76	0.432

Abbreviations: C.I., confidence interval.

**Table 5:** Pearson correlation between differential body mass index and monthly frequency of fish consumption.

Groups	Correlation Test
Fish consumers	r = −0.038
	*p* = 0.574
Type A fish consumption	r = −0.019
	*p* = 0.831
Type B fish consumption	r = −0.222
	*p* = 0.010
Type C fish consumption	r = −0.049
	*p* = 0.557
Type D fish consumption	r = −0.025
	*p* = 0.824

## Data Availability

The data presented in this study are available on request from the corresponding author.
